# State of the Art Review on the Treatment of Psoriatic Disease

**DOI:** 10.31138/mjr.040123.sot

**Published:** 2024-01-31

**Authors:** Eleftherios Pelechas, Evripidis Kaltsonoudis, Michalis P. Migkos, Nikolaos Koletsos, Panagiota G. Karagianni, Alexandros A. Drosos, Paraskevi V. Voulgari

**Affiliations:** 1 Department of Rheumatology, Medical School, University of Ioannina, Ioannina, Greece,; 2 Department of Microbiology, Medical School, University of Ioannina, Ioannina, Greece

**Keywords:** psoriasis, psoriatic arthritis, psoriatic disease, JAK inhibitors, IL inhibitors, TYK inhibitors

## Abstract

**Introduction:**

Psoriasis is an inflammatory skin disease that in some cases is accompanied by systemic manifestations. Given the varied clinical manifestations, the term psoriatic disease probably better reflects the clinical picture of these patients.

**Literature review:**

In most cases, the skin lesions precede joint involvement as well as other potentially involved organs such as the intestine and the eye. Various immune-mediated cellular pathways such as that of TNFα, IL-23, IL-17 as well as other cytokines are involved in the pathophysiology of the psoriatic disease.

**Future insights:**

A better understanding of the way they interfere with our immune system has led to remarkably better disease control and outcomes. This review aims to highlight the newest treatments for psoriatic disease, which are expected to significantly reduce unmet needs and treatment gaps.

## INTRODUCTION

Psoriasis is a chronic inflammatory disease that is characterised by skin lesions which in some cases are accompanied by systemic manifestations. Due to its high heterogeneity, the World Health Organisation (WHO) has classified psoriasis as a serious disease.^1^ It affects 2–3% of the population and presents significant effects on the physical and mental health of the patients.^2,3^ Psoriatic lesions result from an increased proliferation and disturbed differentiation of the keratinocytes.^4^ In the majority of cases, skin lesions precede joint manifestations as well as other organ infestations (bowel, eyes). Given the aforementioned various clinical manifestations, the term psoriatic disease probably reflects in a better manner the whole clinical picture of those affected.^5^ In addition, psoriatic disease may develop a variety of well-known associated comorbidities including cardiovascular disease, obesity and metabolic syndrome, diabetes, osteoporosis, malignancy, fatty liver disease, depression, and anxiety.^6^

Various immune-mediated cellular pathways such as that of TNF-α, IL-23, and IL-17 are involved in the pathophysiology of psoriasis and psoriatic arthritis, and their understanding has led to remarkably better control of it.^4,7^ Nowadays, there are various treatment options that are already approved by the regulatory bodies and rely on blocking those cytokines with good to excellent results so far. The main aim of the so-called targeted treatments with biologics is the long-term modulation of the psoriatic disease, with immediate but also long-term results of the signs and symptoms of the disease including the radiological progression. Finally, there is a growing body of evidence that not only the psoriatic disease in sine gets improved, but also several comorbidities can benefit by the use of these treatments.^8^

Various clinical and genetic phenotypes are involved in psoriasis making the same disease to respond in a different manner when it comes to therapeutic regimens. Moreover, apart from the skin and joints, the targeted therapeutic options are affected by the presence of the comorbidities. On the other hand, it has been shown by different studies that some biomarkers can be used in order to assess the response of the disease but due to different settings they can’t be reliably used at the moment unanimously on an everyday clinical practice.^9^ Thus, the heterogeneity of the disease on one hand and the lack of specific biomarkers on the other hand, lead to a therapeutic ineffectiveness in some instances. One example is this of apremilast, a phosphodiesterase-4 inhibitor, that has significant data on effectiveness and safety for skin psoriasis, but modest results on the polyarticular psoriatic arthritis, and almost no effect on the axial phenotype of the disease.^10^ This is the reason that the European League Against Rheumatism (EULAR) on the last update for the treatment of psoriatic arthritis does not include it as a potent agent as the newer biologic disease-modifying anti-rheumatic drugs (bDMARDs) or the Janus Kinase (JAK) inhibitors.^11^

## CURRENT UNMET NEEDS

Despite the significant progress in the treatment of psoriatic disease (**Table 1**), there is a proportion of patients that do not respond or develop side effects on the available targeted treatments.^12^ In some clinical trials with bDMARDs, it has been reported that up to 40% of the patients are not responding. In fact, in obese patients the percentage is even higher, reaching approximately 50%, when one must have in mind that the psoriatic disease can be affected by emotional instability and stress which is more prevalent in obese people.^13^ On the other hand, obesity is not used as a prognostic factor for treatment response, and this is something that could be used in the future.^14^ Moreover, the phenomenon of the secondary failure in psoriasis treatment is well-established, and it seems that approximately 30% of the patients will discontinue a tumour necrosis factor α (TNFα) agent within one year of treatment. This is even more prevalent in obese women with various comorbidities.^15^

**Table 1.** Overview of biologics and small molecules for the treatment of psoriatic arthritis.

**Drug**	**Structure**	**Mechanism of action**	**Dose**
**Adalimumab**	Fully human IgG1 monoclonal antibody	TNFα inhibitor	40mg subcutaneous every other week
**Certolizumab**	Pegylated humanised antigen-binding fragment (Fab) of an anti-TNF monoclonal antibody	TNFα inhibitor	200mg subcutaneous every other week or 400mg every 4 weeks, post induction
**Etanercept**	Recombinant human fusion protein	Soluble TNF receptor (TNF inhibitor)	50mg subcutaneous weekly
**Golimumab**	Fully human IgG1 monoclonal antibody	TNF inhibitor	50mg subcutaneous monthly
**Infliximab**	Chimeric IgG1 monoclonal antibody	TNF inhibitor	5mg/Kg intravenous every 8 weeks, post induction
**Secukinumab**	Human IgG1 monoclonal antibody	IL-17A inhibitor	150mg subcutaneous monthly, post induction
**Ixekizumab**	Humanised IgG4 monoclonal antibody	IL-17A inhibitor	80mg subcutaneous every 4 weeks, post induction
**Ustekinumab**	Fully human IgG1 monoclonal antibody	p40 subunit of IL-12/IL-23	45mg subcutaneous every 12 weeks, post induction. 90mg used for BW>100Kg.
**Apremilast**	Targeted synthetic agent	PDE4 inhibitor	30mg po BID, post titration
**Tofacitinib**	Targeted synthetic agent	JAK1/JAK3 inhibitor (predominantly)	5mg po BID
**Upadacitinib**	Targeted synthetic agent	JAK1 selective inhibitor	15mg po daily

TNF: tumour necrosis factor; IL: interleukin; PDE: phosphodiesterase; JAK: Janus Kinase; BW: body weight; BID: twice a day.

Real-world data showed that a poor treatment response has a negative impact on the patients’ quality of life regardless of age, sex, smoking habits, body mass index (BMI), and disease duration. In these cases of primary treatment failure, a timely change of treatment to another biologic agent is preferred. On the other hand, despite the low treatment persistence on patients that changed from a TNFα inhibitor to another, this treatment strategy is currently applauded because a percentage of the patients will finally respond.^16^ In addition, despite the newer biologic agents with different mode of action, there is not a significant improvement either, with a similar percentage of patients failing to respond as seen in naïve patients.^16,17^ Thus, an oxymoron is achieved with the treatment strategies. We have better medicines but the patients are undertreated achieving a low disease activity only in 17% of those on conventional synthetic (cs)DMARDs and 57% in those on bDMARDs.^18^ Under these circumstances, a personalised treatment regimen should be applied to these patients using all the available guidelines and treatment algorithms from the EULAR, the American College of Rheumatology (ACR), and the Group for Research and Assessment of Psoriasis and Psoriatic Arthritis (GRAPPA).^11,19–20^ Ideally, treatment strategies should be based on the phenotypic differences of the T-helper cells. This kind of strategies showed significantly higher effectiveness in comparison with the typical treatments with bDMARDs, underlining the importance of precise medicine or personalised medicine strategies.^21^ Since then, and after discussing with the patients, we should focus on patients’ characteristics such as extra-articular manifestations and comorbidities, and choose the right treatment for them.

## PATHOPHYSIOLOGY OF PSORIATIC DISEASE

The discovery of novel drivers of inflammation in psoriatic arthritis (PsA) have led to the identification of IL-17 which is produced by several cells, in addition to CD4+ T-helper (Th17) cells, and which raises the potential for novel pathogenic pathways in psoriasis and the PsA. Traditionally, CD4 and CD8 lymphocytes display pathogenic phenotypes at the sites of disease. The responses of these cells to non-conventional immune stimuli may explain clinical features of these diseases and potential therapeutic mechanisms of therapies such as the JAK inhibitors.^22^ Also, from 2003 to 2016 there was a therapeutic “dominance” of the TNFα inhibitors. But the discovery of the IL-23/IL-17 pathway in 2005, set new therapeutic targets for molecules and signalling pathways. These proved crucial in the pathophysiology of not only axial spondyloarthritis, but also psoriasis and PsA (psoriatic disease). However, the microenvironment of tissue-specific inflammation in each disease differs. IL-17 secretion in the skin appears to be mediated by local IL-23 production, whereas IL-17 production at the entheses (both on axial and peripheral joints) may be independent of IL-23. IL-23 is involved in the Th17 cell differentiation, mediating the conversion of non-pathogenic Th17 cells into pathogenic that can then migrate to local tissues.^23^ Traditionally, it has been thought that a major source of IL-17 is T-cells and that IL-17 production is under the control of IL-23. This seems to have changed today as a number of other cells contribute to its production. In any case, the IL-23 and IL-17 cytokines have an important role both in the pathogenesis and as a therapeutic target and this has been shown in animal models and later in humans as far as it concerns some chronic inflammatory diseases.

## THE TRANSITION OF PSORIASIS TO PSORIATIC ARTHRITIS

It is well documented that, in the vast majority of cases, psoriasis may appear years before joint involvement and a review of twenty epidemiologic studies found that the reported proportion of psoriatic arthritis among psoriasis patients ranges from 7–26%.^24,25^ During this time, various environmental, microbial and genetic factors contribute to the transition of inflammation from the skin to the joints.^26^ Some of the known risk factors are severe psoriasis,^27^ onychopsoriasis,^28^ obesity,^29^ smoking,^30^ and alcohol.^31^ Since no diagnostic criteria or specific tests are available, diagnosis is usually based on the identification of inflammatory musculoskeletal features in the joints, and the presence of psoriasis of the skin and/or nails (onychopsoriasis). Based on current therapeutic practice, the initiation of medication is done in the established disease (CASPAR criteria), i.e. where there is clinical symptomatology and/or imaging evidence. But, having in mind that there is a better understanding of the disease in a molecular basis we need to explore more in the direction of developing new methods in order to start treatment at a preclinical stage. This may significantly improve both short-term and long-term outcomes, by improving musculoskeletal and skin manifestations, as well as reducing the radiographic damage.

## NEW BIOLOGIC TREATMENTS

Newer available biologic therapies (**Table 2**) have been studied and their arrival may, and is expected, to fill an important therapeutic gap.

**Table 2.** Latest agents for the treatment of psoriatic arthritis.

**JAK inhibitors**	**TYK2 inhibitors**	**IL-17 inhibitors**	**IL-23 inhibitors**
Tofacitinib (JAK1/3)	Deucravacitinib	Bimekizumab (IL-17A/F)	Guselkumab
Upadacitinib (JAK1)		Brodalumab (IL-17A/E/F)	Tildrakizumab
Filgotinib (JAK1)			Risankizumab

JAK: Janus Kinase; TYK: tyrosine kinase; IL: interleukin.

### JAK inhibitors

The Janus Kinase (JAK) inhibitors are small molecules of great importance since blockade of the JAK kinase receptor downregulates the production of cytokines that are important in the pathogenesis of PsA. Tofacitinib, an orally administered inhibitor (mainly JAK1 and JAK3), has shown its efficacy in multiple levels of disease activity, and after two pivotal phase 3 trials, it has been approved in combination with methotrexate (MTX) for the treatment of PsA.^32^ Upadacitinib is a more selective JAK inhibitor (JAK1), which through two pivotal phase 3 trials, (SELECT-PsA 1 and SELECT-PsA 2) has demonstrated efficacy in multiple levels of disease activity.^33,34^ The extension of these studies showed sustained efficacy at 56 weeks. The safety profile of upadacitinib was consistent with previously reported results in all indications, with no new safety signals. These two studies approved the drug at a dosage scheme of 15mg per day for patients with PsA refractory to treatment with csDMARDs. Filgotinib (200mg per day), also a selective oral JAK 1 inhibitor, was evaluated in a phase 2 study (EQUATOR).^35^ It included 131 patients with failure to control the disease on csDMARDs and was compared with a placebo group. Response was seen rapidly from week 1, while adverse events were similar between the two groups. There have been concerns about testicular toxicity, which is being studied in the MANTA-RAy study, the results of which are eagerly awaited.

Finally, review and meta-analysis of the results of JAK inhibitors (as far as it concerns their safety and efficacy) with five randomised controlled trials (RCTs) of 3293 PsA patients treated with different JAK inhibitors of placebo (2 phase III studies for tofacitinib, 1 phase II study for filgotinib and 2 phase III studies for upadacitinib), demonstrated a statistically significant benefit of JAK inhibitors over placebo in terms of efficacy without emerging any new safety signals.

### TYK inhibitors

TYK2 inhibitors differ from JAK inhibitors in binding to the active site in the kinase domain. It is an intracellular kinase that mediates IL-23, IL-12, and interferon α/β. Data from a phase II study for deucravacitinib, showed that it was effective for the treatment of active PsA.^36^ Both 6mg and 12mg dosage schemes, showed a significant ACR20 response at week 16 compared to placebo (52,9%, 62,7%, and 31,8% respectively). It was also effective in several secondary endpoints, including ACR50/70 and enthesitis, with no safety issues related to venous thromboembolism or hematologic abnormalities. Other therapies under clinical evaluation for the treatment of psoriasis are the retinoic acid-related nuclear receptor RORγt inhibitor, which is a master regulator of Th17 cells.

### IL-23 inhibitors

IL-23 is believed to be a key regulatory cytokine in the pathogenesis of PsA, and targeting it appears to bring about many of the expected therapeutic effects. Guselkumab, tildrakizumab and risankizumab are three IL-23 inhibitors approved by the European Medicines Agency (EMA) and the Food and Drug Administration (FDA) for the treatment of moderate to severe plaque psoriasis in adult patients (**Table 3**). By binding to the p19 subunit, guselkumab blocks the binding of extra-cellular IL-23 to the IL-23 cell surface receptor, thereby inhibiting IL-23-mediated intracellular signalling, activation, and cytokine production. In a phase 3 study (DISCOVER-1), patients received guselkumab every 4 or every 8 weeks.^37^ Response rates by ACR20 at week 24 were significantly higher for guselkumab every 4 or every 8 weeks than in placebo (59,4% and 52,0% versus 22,2% respectively). In DISCOVER-2, a larger study in biologic-naïve patients, guselkumab was administered every 4 or every 8 weeks versus placebo.^38^ ACR20 response at week 24 was 63,7% and 64,1% versus 32,9% respectively. The response was clearly greater than in DISCOVER-1, which also included patients with failure of even two TNFα inhibitors. The difference between drug and placebo was also significant in secondary endpoints including radiological progression, enthesitis, and dactylitis. The observed safety profile was similar to previous studies in patients with psoriasis. A meta-analysis comparing guselkumab with targeted therapies in PsA regarding safety and efficacy in joint and skin lesions showed that guselkumab had a very good efficacy in arthritis, (comparable to IL-17A inhibitors and TNFα inhibitors), while providing a better PASI response than many other treatments.^39^

**Table 3.** Overview of latest approved IL-23 inhibitors for the treatment of psoriasis.

**Drug**	**Structure**	**Mechanism of action**	**Dose**
**Guselkumab**	Human IgG1λ monoclonal antibody	p19 subunit of IL-23	100mg subcutaneous every 8 weeks, post induction
**Tildrakizumab**	Humanised IgG1κ monoclonal antibody	p19 subunit of IL-23	100mg subcutaneous every 12 weeks, post induction
**Risankizumab**	Humanised IgG1κ monoclonal antibody	p19 subunit of IL-23	150mg subcutaneous every 12 weeks, post induction

IL: interleukin.

Tildrakizumab, also approved for the treatment of moderate to severe plaque psoriasis is under investigation for the treatment of PsA. In a 2b phase study at week 24, ACR20 response was observed in 71,4–79,5% of tildrakizumab patients versus 50,6% of placebo patients.^40^ At the same time, phase 3 studies are being conducted to evaluate its effectiveness in PsA. Finally, risankizumab (150mg) also an approved monoclonal antibody against interleukin-23p19 was evaluated in PsA.^41^ ACR20 response for risankizumab was 57% and 51% vs 34% and 27% for placebo at week 24 with the known safety profile of risankizumab observed in psoriasis. A meta-analysis of clinical trials comparing the safety and benefit-risk profile of biologic and oral therapies in patients with moderate to severe plaque psoriasis showed that anti-IL-23 agents were associated with low rates of adverse events, and that risankizumab had the most favourable long-term benefit-risk profile.^42^ Of note that, IL-23 inhibitors have not shown a good response to axial disease.

### IL-17 Inhibitors

Secukinumab, an IL-17A inhibitor, vs adalimumab, a TNFα inhibitor with recognised efficacy and safety in psoriasis, was compared in EXCEED study (a double-blind, randomised, phase 3b trial) in patients with active PsA. In this study secukinumab did not demonstrate superiority (no statistical significance in the primary endpoint of ACR20 response at week 52), but showed higher treatment retention than adalimumab. The conclusions of such comparative studies of two biological agents with different mechanisms of action, could help in making clinical decisions in the management of PsA.^43^

Ixekizumab, another IL-17A inhibitor, in a 24-week phase 3 trial (SPIRIT-P2) in patients with active PsA who had failed after treatment of TNFα inhibitors showed that both the 2-week and 4-week regimens improved signs and symptoms in active PsA, ACR20 with ixekizumab every 4 weeks [65 (53%) patients, effect size vs placebo 33,8% (95% CI 22,4–45,2), p<0,0001)] and ixekizumab every 2 weeks [59 (48%) patients, 28,5% (17,1–39,8), p<0,0001) versus placebo patients [23 (20%) patients] with a safety profile consistent with previous studies.^44^ IL-17 inhibitors have shown also a good clinical response to axial disease as well as the IL-17A-F inhibitors.

### IL-17A-F inhibitors

IL-17A and IL-17F appear to act synergistically in pathological bone formation, thus suggesting that neutralisation of both cytokines inhibits this process in a better manner than inhibition of IL-17A alone. Bimekizumab in a phase 2b study (BEACTIVE) with 206 patients showed a good efficacy and safety profile in two dosage regimens of 16mg and 160mg (with or without a loading dose of 320mg).^45^ The onset of action was rapid with ACR50 response maintained at 48 weeks, and results extended to 152 weeks.^46^ Brodalumab, a human IgG2 monoclonal antibody binds with high affinity to the IL-17 receptor (R) and inhibits IL-17A, IL-17E and IL-17F. The AMVISION-1 and AMVISION-2, two randomised phase III trials compared brodalumab with placebo (brodalumab 140mg or 210mg at weeks 0, 1, and every 2 weeks to 24 weeks) in 962 patients. The ACR20 response rates at week 16 in both brodalumab treatment groups were 45,8% and 47,9% for 140mg and 210mg respectively versus placebo 20,9% (p<0,0001). Similar results were seen at week 24. Significantly higher percentages of brodalumab-treated patients also achieved secondary endpoints ACR50/70. Finally, brodalumab was well tolerated with a safety profile consistent with the other IL-17 inhibitors.^47^

## FUTURE TREATMENT INSIGHTS

Although the therapeutic armamentarium for the treatment of psoriasis and PsA has expanded significantly over the past thirty years, additional drugs are needed for optimal disease care. Based on efficacy and safety, biologics targeting the IL-23 and IL-17 pathways represent one of the greatest achievements of dermatology in the past decade. In many patients, there may be little or no relationship between the severity of musculoskeletal inflammation and the severity of skin or nail psoriasis. The reason for the heterogeneity of this disease can be explained by differences in different genotypes, especially in the HLA region. New targeted therapies for PsA have been approved and additional therapies are under development. These advances have significantly improved both short- and long-term outcomes, including improvement in musculoskeletal and skin manifestations as well as the reduction of radiographic damage. In the coming years, it needs to be determined which of the modern treatments, including the well-established TNFα therapy, most effectively reduces and/or reverses the comorbidi-ties of psoriatic disease, with an emphasis on metabolic and cardiovascular ones. There is reasonable hope that early use of specific therapies not only eliminates short-lived pathogenic cells but also prevents the emergence and expansion of long-lived pathogenic cells. It is also possible that the patients being just a “cohort number”, may not be considered equally in all studies as far as it concerns sample time, patient age, disease duration and disease severity. This is probably the reason why drugs with the same and/or similar mechanism of action lead to ambiguous results. It also highlights the challenges facing researchers seeking to characterise the pathogenesis of complex autoimmune diseases.

A systematic review and meta-analysis compared treatments for the effectiveness of arthritis (ARC response), psoriasis (PASI), enthesitis, and dactylitis. It also assessed the safety of the drugs on the basis of discontinuation due to adverse effects. It included a total of 46 studies. The results showed that some TNFα inhibitors performed numerically, but without statistical significance, better results than IL-inhibitors in ACR response, but had a worse PASI response. Guselkumab and the IL-17A or IL-17R inhibitors (brodalumab, ixekizumab, secukinumab) were the best in PASI response. IL-inhibitors and adalimumab were equally effective in resolving enthesitis and dactylitis. Infliximab with or without methotrexate, certolizumab 400mg every 4 weeks and tildrakizumab had the highest rates of adverse events. The conclusion was that IL-17A & IL-17R inhibitors and guselkumab offered better efficacy than TNFα inhibitors in the cutaneous manifestations, enthesitis and dactylitis and similar efficacy in ACR response.

## CONCLUSIONS

During the last decades, significant advances have been made in the understanding and treatment of psoriatic disease (psoriasis and PsA). However, there is currently no method for predicting the optimal therapeutic strategy, both for well-established and emerging therapies. The choice of drug for the treatment of psoriatic disease should be based on the predominant clinical phenotype, and treatment should be initiated very close to the point of onset of inflammation.

## References

[1] World Health Organization psoriasis. Global report on. Glob Rep Psoriasis [Internet]. 2016;978. Available from: http://www.who.int/about/licensing/copyright_form/index.html%0Ahttp://www.who.int/about/licensing/

[2] Pelechas E Kaltsonoudis E Voulgari PV Drosos AA. Psoriatic Arthritis. In: Illustrated Handbook of Rheumatic and Musculo-Skeletal Diseases. Springer, Cham, 2019. 10.1007/978-3-030-03664-5_5

[3] Alamanos Y Pelechas E Voulgari PV Drosos AA. Incidence of spondyloarthritis and its subtypes: a systematic review. Clin Exp Rheumatol 2021;39(3):660–7. doi: 10.55563/clinexperheumatol/yycy0o. 32896268

[4] Ritchlin CT Colbert RA Gladman DD. Psoriatic Arthritis. N Engl J Med.2017;376(10):957–70. 28273019 10.1056/NEJMra1505557

[5] Cigolini C Fattorini F Gentileschi S Terenzi R Carli L. Psoriatic arthritis: one year in review 2022. Clin Exp Rheumatol 2022;40(9). 10.55563/clinexprheumatol/x3sfxe 36129799

[6] Fragoulis GE Evangelatos G Tentolouris N Fragkiadaki K Panopoulos S Konstantonis G Higher depression rates and similar cardiovascular comorbidity in psoriatic arthritis compared with rheumatoid arthritis and diabetes mellitus. Ther Adv Musculoskelet Dis 2020;12:1759720X20976975. doi: 10.1177/1759720X20976975 PMC7727079 33343726

[7] Mantravadi S Ogdie A Kraft WK. Tumor necrosis factor inhibitors in psoriatic arthritis. Expert Rev Clin Pharmacol 2017;10(8):899–910. 28490202 10.1080/17512433.2017.1329009 PMC6348387

[8] Gialouri CG Evangelatos G Fragoulis GE. Choosing the appropriate target for the treatment of psoriatic arthritis: TNFα, IL-17, IL-23 or JAK inhibitors? Mediterr J Rheumatol 2022;33(Suppl 1):150–61. 36127928 10.31138/mjr.33.1.150 PMC9450184

[9] Magee C Jethwa H FitzGerald OM Jadon DR. Biomarkers predictive of treatment response in psoriasis and psoriatic arthritis: a systematic review. Ther Adv Musculoskelet Dis 2021;13. 10.1177/1759720X211014010 PMC8111521 33995606

[10] Schett G. Apremilast in psoriatic arthritis. Clin Exp Rheumatol 2015;33(5 Suppl 93):S98–100. 26472278

[11] Gossec L Baraliakos X Kerschbaumer A De Wit M McInnes I Dougados M EULAR recommendations for the management of psoriatic arthritis with pharmacological therapies: 2019 update. Ann Rheum Dis 2020;79(6):S700–12. 10.1136/annrheumdis-2020-217159 PMC7286048 32434812

[12] Tahir H Grewal S. Current unmet needs and emerging novel pharmacotherapies in psoriatic arthritis. Expert Opin Pharmacother 2022;23(4):417–20. 34787511 10.1080/14656566.2021.2006184

[13] Gialouri CG Evangelatos G Zhao SS Kouna K Karamanakos A Iliopoulos A Depression and anxiety in a real-world psoriatic arthritis longitudinal study: should we focus more on patients’ perception? Clin Exp Rheumatol 2023;41(1):159–65. 35819812 10.55563/clinexprheumatol/8qxo80

[14] Singh S Facciorusso A Singh AG Vande Casteele N Zarrinpar A Prokop LJ Obesity and response to anti-tumor necrosis factor-α agents in patients with select immune-mediated inflammatory diseases: A systematic review and meta-analysis. PLoS One 2018;13(5). 10.1371/journal.pone.0195123 PMC5957395 29771924

[15] Stober C Ye W Guruparan T Htut E Clunie G Jadon D. Prevalence and predictors of tumour necrosis factor inhibitor persistence in psoriatic arthritis. Rheumatology (Oxford) 2018;57(1):158–63. 29077973 10.1093/rheumatology/kex387

[16] Costa L Perricone C Chimenti MS Puente AD Caso P Peluso R Switching between biological treatments in psoriatic arthritis: a review of the evidence. Drugs R D 2017;17(4):509–522. 29058302 10.1007/s40268-017-0215-7 PMC5694428

[17] Alten R Conaghan PG Strand V Sullivan E Blackburn S Tian H Unmet needs in psoriatic arthritis patients receiving immunomodulatory therapy: results from a large multinational real-world study. Clin Rheumatol 2019;38(6):1615–26. 30719594 10.1007/s10067-019-04446-z

[18] Ariani A Santilli D Mozzani F Lumetti F Lucchini G Di Donato E Cycling or swap biologics and small molecules in psoriatic arthritis: Observations from a real-life single center cohort. Medicine (Baltimore) 2021;100(16):e25300. 33879661 10.1097/MD.0000000000025300 PMC8078287

[19] Zardin-Moraes M Da Silva ALFA Saldanha C Kohem CL Coates LC Henrique LR Prevalence of Psoriatic Arthritis Patients Achieving Minimal Disease Activity in Real-world Studies and Randomized Clinical Trials: Systematic Review with Metaanalysis. J Rheumatol 2020;47(6):839–46. 31575702 10.3899/jrheum.190677

[20] Singh JA Guyatt G Ogdie A Gladman DD Deal C Deodhar A Special Article: 2018 American College of Rheumatology/National Psoriasis Foundation Guideline for the Treatment of Psoriatic Arthritis. Arthritis Care Res 2019;71(1):2–29. 10.1002/acr.23789 PMC8265826 30499259

[21] Coates LC Corp N van der Windt DA Soriano ER Kavanaugh A. GRAPPA Treatment Recommendations: An Update From the 2020 GRAPPA Annual Meeting. J Rheumatol 2021;97:65–6. 10.3899/jrheum.201681 34074672

[22] Miyagawa I Nakayamada S Nakano K Kubo S Iwata S Miyazaki Y Precision medicine using different biological DMARDs based on characteristic phenotypes of peripheral T helper cells in psoriatic arthritis. Rheumatology (Oxford) 2019;58(2):336–44. 29618121 10.1093/rheumatology/key069

[23] O’Brien-Gore C Gray EH Durham LE Taams LS Kirkham BW. Drivers of Inflammation in Psoriatic Arthritis: the Old and the New. Curr Rheumatol Rep 2021;23(6). 10.1007/s11926-021-01005-x 33909160

[24] Busse K Liao W. Which psoriasis patients develop psoriatic arthritis? Psoriasis Forum 2010;16(4):17–25 25346592 PMC4206220

[25] Prey S Paul C Bronsard V Puzenat E Gourraud PA Aractingi S Assessment of risk of psoriatic arthritis in patients with plaque psoriasis: a systematic review of the literature. J Eur Acad Dermatol Venereol 2010;24 Suppl 2:31–5. 20443998 10.1111/j.1468-3083.2009.03565.x

[26] Sieper J Poddubnyy D Miossec P. The IL-23-IL-17 pathway as a therapeutic target in axial spondyloarthritis. Nat Rev Rheumatol 2019;15(12):747–57. 31551538 10.1038/s41584-019-0294-7

[27] Haroon M Kirby B FitzGerald O. High prevalence of psoriatic arthritis in patients with severe psoriasis with suboptimal performance of screening questionnaires. Ann Rheum Dis 2013;72(5):736–740. 22730367 10.1136/annrheumdis-2012-201706

[28] Wilson F Icen M Crowson C McEvoy M Gabriel S Kremers H. Incidence and clinical predictors of psoriatic arthritis in patients with psoriasis: a population-based study. Arthritis Rheum 2009;61(2):233–239. 19177544 10.1002/art.24172 PMC3061343

[29] Soltani-Arabshahi R Wong B Feng B Goldgar D Duffin K Krueger G. Obesity in early adulthood as a risk factor for psoriatic arthritis. Arch Dermatol 2010;146(7):721–726). 20644032 10.1001/archdermatol.2010.141

[30] Eder L Law T Chandran V Shanmugarajah S Shen H Rosen CF Association between environmental factors and onset of psoriatic arthritis in patients with psoriasis. Arthritis Care Res (Hoboken) 2011;63(8):1091–7. 21560259 10.1002/acr.20496

[31] Wu S Cho E Li WQ Han J Qureshi AA. Alcohol intake and risk of incident psoriatic arthritis in women. J Rheumatol 2015;42(5):835–40. 25834201 10.3899/jrheum.140808 PMC4600066

[32] Mease P Hall S FitzGerald O van der Heijde D Merola JF Avila-Zapata F Tofacitinib or Adalimumab versus Placebo for Psoriatic Arthritis. N Engl J Med 2017;377(16):1537–50. 29045212 10.1056/NEJMoa1615975

[33] McInnes IB Anderson JK Magrey M Merola JF Liu Y Kishimoto M Trial of Upadacitinib and Adalimumab for Psoriatic Arthritis. N Engl J Med 2021;384(13):1227–39. 33789011 10.1056/NEJMoa2022516

[34] Mease PJ Lertratanakul A Anderson JK Papp K Van Den Bosch F Tsuji S Upadacitinib for psoriatic arthritis refractory to biologics: SELECT-PsA 2. Ann Rheum Dis 2021;80(3):312–20. 33272960 10.1136/annrheumdis-2020-218870 PMC7892371

[35] Mease P Coates LC Helliwell PS Stanislavchuk M Rychlewska-Hanczewska A Dudek A Efficacy and safety of filgotinib, a selective Janus kinase 1 inhibitor, in patients with active psoriatic arthritis (EQUATOR): results from a randomised, placebo-controlled, phase 2 trial. Lancet (London, England) 2018;392(10162):2367–77. 30360969 10.1016/S0140-6736(18)32483-8

[36] Mease PJ Deodhar AA Van Der Heijde D Behrens F Kivitz AJ Neal J Efficacy and safety of selective TYK2 inhibitor, deucravacitinib, in a phase II trial in psoriatic arthritis. Ann Rheum Dis. 2022;81(6):815–22. 35241426 10.1136/annrheumdis-2021-221664 PMC9120409

[37] Deodhar A Helliwell PS Boehncke WH Kollmeier AP Hsia EC Subramanian RA Guselkumab in patients with active psoriatic arthritis who were biologic-naive or had previously received TNFα inhibitor treatment (DISCOVER-1): a double-blind, randomised, placebo-controlled phase 3 trial. Lancet (London, England) 2020;395(10230):1115–25. 32178765 10.1016/S0140-6736(20)30265-8

[38] Mease PJ Rahman P Gottlieb AB Kollmeier AP Hsia EC Xu XL Guselkumab in biologic-naive patients with active psoriatic arthritis (DISCOVER-2): a double-blind, randomised, placebo-controlled phase 3 trial. Lancet (London, England) 2020;395(10230):1126–36. 32178766 10.1016/S0140-6736(20)30263-4

[39] Mease PJ McInnes IB Tam LS Eaton K Peterson S Schubert A Comparative effectiveness of guselkumab in psoriatic arthritis: results from systematic literature review and network meta-analysis. Rheumatology (Oxford) 2021;60(5):2109–21. 33844022 10.1093/rheumatology/keab119 PMC8121447

[40] Mease PJ Chohan S Fructuoso FJG Luggen ME Rahman P Raychaudhuri SP Efficacy and safety of tildrakizumab in patients with active psoriatic arthritis: results of a randomised, double-blind, placebo-controlled, multiple-dose, 52-week phase IIb study. Ann Rheum Dis 2021;80(9):1147–57. 33985942 10.1136/annrheumdis-2020-219014 PMC8372392

[41] Kristensen LE Keiserman M Papp K McCasland L White D Lu W Efficacy and safety of risankizumab for active psoriatic arthritis: 24-week results from the randomised, double-blind, phase 3 KEEPsAKE 1 trial. Ann Rheum Dis 2022;81(2):225–31. 34911706 10.1136/annrheumdis-2021-221019 PMC8762015

[42] Shear NH Betts KA Soliman AM Joshi A Wang Y Zhao J Comparative safety and benefit-risk profile of biologics and oral treatment for moderate-to-severe plaque psoriasis: A network meta-analysis of clinical trial data. J Am Acad Dermatol 2021;85(3):572–81. 33631216 10.1016/j.jaad.2021.02.057

[43] McInnes IB Behrens F Mease PJ Kavanaugh A Ritchlin C Nash P Secukinumab versus adalimumab for treatment of active psoriatic arthritis (EXCEED): a double-blind, parallel-group, randomised, active-controlled, phase 3b trial. Lancet (London, England) 2020;395(10235):1496–505. 32386593 10.1016/S0140-6736(20)30564-X

[44] Nash P Kirkham B Okada M Rahman P Combe B Burmester GR Ixekizumab for the treatment of patients with active psoriatic arthritis and an inadequate response to tumour necrosis factor inhibitors: results from the 24-week randomised, double-blind, placebo-controlled period of the SPIRIT-P2 phase 3 trial. Lancet 2017;389(10086):2317–27. 28551073 10.1016/S0140-6736(17)31429-0

[45] Coates LC McInnes IB Merola JF Warren RB Kavanaugh A Gottlieb AB Safety and Efficacy of Bimekizumab in Patients with Active Psoriatic Arthritis: 3-Year Results from a Phase 2b Randomized Controlled Trial and its Open-Label Extension Study. Arthritis Rheumatol (Hoboken, NJ) 2022; 10.1002/art.42280 PMC10100448 35829656

[46] Ritchlin CT Kavanaugh A Merola JF Schett G Scher JU Warren RB Bimekizumab in patients with active psoriatic arthritis: results from a 48-week, randomised, double-blind, placebo-controlled, dose-ranging phase 2b trial. Lancet (London, England) 2020;395(10222):427–40. 32035552 10.1016/S0140-6736(19)33161-7

[47] Mease PJ Helliwell PS Hjuler KF Raymond K Mcinnes I. Brodalumab in psoriatic arthritis: results from the randomised phase III AMVISION-1 and AMVISION-2 trials. Ann Rheum Dis 2021;80(2):185–93. 33106286 10.1136/annrheumdis-2019-216835 PMC7815636

